# Peripheral blood cell count ratios are predictive biomarkers of clinical response and prognosis for non‐surgical esophageal squamous cell carcinoma patients treated with radiotherapy

**DOI:** 10.1002/jcla.23468

**Published:** 2020-07-17

**Authors:** Xiaohui Zhi, Kan Jiang, Yue Shen, Xinyu Su, Ke Wang, Yuanyuan Ma, Liqing Zhou

**Affiliations:** ^1^ Department of Radiation Oncology the Affiliated Huai’an Hospital of Xuzhou Medical University the Second People’s Hospital of Huai’an Huai’an China; ^2^ Shandong Provincial Key Laboratory of Radiation Oncology Cancer Research Center Shandong Academy of Medical Sciences Shandong Cancer Hospital affiliated to Shandong University Jinan China

**Keywords:** clinical response, esophageal squamous cell carcinoma, lymphocyte‐to‐monocyte ratio, neutrophil‐to‐lymphocyte ratio, platelet‐to‐lymphocyte ratio, prognosis, radiotherapy

## Abstract

**Background:**

Peripheral blood cell count ratios, including the neutrophil‐to‐lymphocyte ratio (NLR), platelet‐to‐lymphocyte ratio (PLR), and lymphocyte‐to‐monocyte ratio (LMR), have been reported to be prognostic factors in many malignancies as markers of inflammation and immune status. The aim of this study was to determine whether NLR, PLR, or LMR can be clinical response and prognostic biomarkers of non‐surgical esophageal squamous cell carcinoma (ESCC) patients treated with radiotherapy.

**Methods:**

193 non‐surgical ESCC patients who underwent radiotherapy were retrospectively analyzed. The peripheral blood cell count ratios were obtained before, during (weekly) and at the end of the treatment. Then, we compared the subsequent results with the corresponding pretreatment values and computed the rates of change, which were defined as cNLR, cPLR, and cLMR. Univariate and multivariate Cox regression analyses were used for overall survival (OS). Ordinal logistic regression was used to analyze the clinical response.

**Results:**

In multivariate analysis, cNLR at week 4(*P *= .026) and week 5(*P *= .025) during radiotherapy were significantly associated with OS, along with BMI, tumor stage, tumor length, tumor location, and grade of adverse events. Besides, BMI, tumor stage, tumor length, adverse event grade, cNLR at week 4(*P *= .044) and week 5(*P *= .013), and cPLR at week 4(*P *= .034) and week 5(*P *= .015) were significantly associated with the clinical response in the multivariate logistic regression analysis.

**Conclusions:**

The cNLR at weeks 4 and 5 was negatively correlated with the OS and clinical response of non‐surgical ESCC patients treated with radiotherapy. The elevated cPLR at weeks 4 and 5 was only related to poor clinical response.

## Graphical abstract

In this retrospective study, we demonstrated that the cNLR at weeks 4 and 5 during RT had a significant negative correlation with the OS and clinical responses of non‐surgical ESCC patients treated with radiotherapy, while an elevated cPLR at weeks 4 and 5 was only related to poor clinical response. During radiotherapy, the median cNLR and cPLR increased gradually every week, reaching the highest values at week 5 and then declining. Conversely, cLMR decreased to the minimum value at week 5 but had no predictive ability for either OS or clinical response.

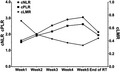

## 1 INTRODUCTION

Esophageal carcinoma (EC) is one of the most common cancers worldwide. The incidence of EC is ranked seventh among all types of malignant tumors, and mortality is ranked sixth, according to Global Cancer Statistics 2018.^1^ The 5‐year age‐standardized net survival of EC is approximately 10%–30% worldwide.^2^ Eastern Asia, including China, has a high incidence of EC. Our research group is located in one of the highest incidence areas of EC in China. The major histological type of EC in China is esophageal squamous cell carcinoma (ESCC), which differs from that in Western countries.^3^ At present, the diagnosis and prognosis evaluation of ESCC mostly depend on endoscopic procedures and imaging tests, such as gastroscopy, computed tomography (CT), and esophagram. Unfortunately, unlike other digestive system neoplasms, EC lacks blood biomarkers for predicting prognosis and tumor response to treatment and biomarkers for risk stratification.

Evidence has increasingly shown that inflammation plays an important role in tumor development and progression by modifying the tumor microenvironment.^4^, ^5^, ^6^ Recently, various studies have shifted their sights to inflammatory biomarkers in peripheral blood. Neutrophil and platelet regularly change with the level of systemic inflammation, while lymphocyte and monocyte can indicate the level of immunity. Therefore, peripheral blood cell count ratios, including the neutrophil‐to‐lymphocyte ratio (NLR), platelet‐to‐lymphocyte ratio (PLR), and lymphocyte‐to‐monocyte ratio (LMR), can quantify the inflammatory and immune response. They can be used as practical prognostic biomarkers to evaluate patient outcomes in various malignancies.^7^, ^8^, ^9^ In EC, many researches have estimated the prognostic significance of pretreatment peripheral blood cell count ratios.^10^, ^11^ These shown that the baseline level of inflammation is associated with the prognosis of ESCC patients. However, the existing correlational studies mostly focus on patients undergoing surgery or neoadjuvant therapy. Because of the atypical manifestations in the early stages or intolerance of surgery, a number of ESCC patients have lost the opportunity for surgical therapy by the time their cancers are detected. Given these conditions, radiotherapy (RT) significantly improves the survival rate of non‐surgical ESCC patients.

Therefore, we performed this retrospective study to evaluate whether these simple and repeatable parameters can be clinical response and prognostic biomarkers of non‐surgical ESCC patients treated with radiotherapy.

## 2 MATERIALS AND METHODS

### 2.1 Patient eligibility

A total of 755 patients diagnosed with ESCC by pathology underwent radiotherapy at our institute between January 2013 and December 2016. Patients were omitted from this study if they met any of the following exclusion criteria: (a) patients without records of peripheral blood cell counts before or during the study period; (b) patients with any other malignancy before or during the study; (c) patients who had received previous chemotherapy or radiotherapy; (d) patients who had undergone previous cancer‐related surgery; (e) patients who received palliative or supportive treatment only; (f) patients with underlying diseases that might influence peripheral blood cell counts, such as liver cirrhosis or infection; (g) patients who received certain medications within 5 days before blood sample collection, such as granulocyte colony‐stimulating factor or thrombopoietin; and (h) patients lost to follow‐up. Through the above filters, 193 patients were selected. In addition to the overall analysis of all patients, we conducted subgroup analyses for patients who received concurrent chemoradiotherapy (CCRT) and RT alone. This study was approved by the Ethics Committee of the Affiliated Huai'an Hospital of Xuzhou Medical University.

### 2.2 Clinicopathological data

Pretreatment tumor stage was classified based on the Clinical Classification of Esophageal Carcinoma Treated by Non‐surgical Methods, the accuracy of which has been well confirmed.^12^, ^13^ According to this classification system, T stage was assessed based on tumor length measured by barium esophagram, the maximum esophageal diameter of the largest esophageal lesion on CT scans and whether the tumor invaded adjacent organs. N stage was evaluated based on the diameter and location of the lymph nodes on CT scans. All clinical characteristics were extracted from electronic medical records.

### 2.3 Treatment protocol

All patients underwent CT simulations before radiotherapy. The radiation range involved the primary tumor along with the prophylactic regional lymph nodes. The radiation treatment was delivered as intensity‐modulated radiation treatment (IMRT) using conventional fractionation (CF), simultaneous integrated boost (SIB), or sequential boost (SB). A total radiation dose of 58‐64 Gy (1.8‐2.2 Gy/day, 5 days/week) was given. Before treatment, we carefully evaluated patients’ performance status, organ function, and comorbidities. Radical radiotherapy was administered to the following patients: elderly patients with early stage; patients who were assessed to be unable to tolerate CCRT; and patients who refused chemotherapy. CCRT was recommended for patients with advanced stage disease, normal cardiac function, normal hepatorenal function, and performance status score ≤ 2. According to the Clinical Practice Guidelines for the Diagnosis and Treatment of Esophageal Cancer (version 2), the standard chemotherapy regimen used was TP (Paclitaxel and Cisplatin) or PF (5‐Fluorouracil and Cisplatin). The chemotherapy was given on the first and 22nd day of RT. Two cycles of chemotherapy were completed during RT.

### 2.4 Peripheral blood cell count ratios

Laboratory data were extracted from the electronic medical records. Blood samples were obtained before, during(weekly), and at the end of RT and were analyzed at the clinical laboratory of our institute. The NLR was calculated as the absolute neutrophil count divided by the absolute lymphocyte count. The LMR was indicated as the absolute lymphocyte count divided by the absolute monocyte count. The PLR was calculated by dividing the absolute platelet count by the absolute lymphocyte count. Each patient's peripheral blood cell count ratio prior to treatment was defined as the baseline value. Then, we compared the subsequent results with the baseline and computed the rates of change. These rates were defined as cNLR, cPLR, and cLMR for the NLR, PLR, and LMR, respectively. The formulas were as follows:

$$\text{cNLR}\;\text{at}\;\text{week}\;n=\frac{\text{neutrophil at week n}}{\text{lymphocyte at week n}}/\frac{\text{neutrophil before treatment}}{\text{lymphocyte before treatment}}$$

$$\text{cPLR}\;\text{at}\;\text{week}\;n=\frac{\text{platelet at week n}}{\text{lymphocyte at week n}}/\frac{\text{platelet before treatment}}{\text{lymphocyte before treatment}}$$

$$\text{cLMR}\;\text{at}\;\text{week}\;n=\frac{\text{lymphocyte at week n}}{\text{monocyte at week n}}/\frac{\text{lymphocyte before treatment}}{\text{monocyte before treatment}}$$

### 2.5 Response evaluation and follow‐up

Clinical responses were assessed by barium radiography and CT‐based short‐term outcome evaluation criteria in EC.^14^ Accordingly, the responses of the primary lesion, estimated by barium radiography, were classified into three levels as follows: (a) esophagram complete response: the tumor disappeared completely, the esophagus was smooth and neat, and the mucosa returned to normal; (b) esophagram no response: no significant changes in the tumor, presence of irregular filling defect, and strictures were seen; and (c) esophagram partial response: other responses than those listed above. Thereafter, CT was used to measure the maximum thickness of the esophageal wall and the short‐axis diameter of residual lymph nodes. Then, combined with the results of barium radiography evaluation, the clinical responses were eventually classified into three categories: (a) clinical complete response (cCR): esophagram complete response, maximum thickness of esophageal wall ≤ 1.2 cm, short‐axis diameter of residual lymph nodes ≤ 1.0 cm, and no distant metastasis; (b) clinical no response (cNR): esophagram no response or distant metastasis; and (c) clinical partial response (cPR): other responses than those listed above. Both examinations were conducted 1‐3 months after the completion of RT.

All patients were followed up every 2 or 3 months for the first 2 years by regular phone calls and then every 6 months until April 2019 or until death. Adverse events were evaluated according to the National Cancer Institute's Common Terminology Criteria for Adverse Events (CTCAE v4.03).

### 2.6 Statistical analysis

Univariate and multivariate ordinal logistic regression analyses were performed to assess the association between variables and clinical responses. Independent prognostic factors for OS were determined by univariate and multivariate Cox proportional regression models. To assess the differences between the baseline characteristics of the CCRT and RT alone groups, the chi‐squared test or Fisher's exact test was used for categorical variables, and two independent samples t tests were applied to continuous variables. We estimated the OS of patients in each group by the Kaplan‐Meier method and applied log‐rank tests to compare the survival curves. Variance analysis or the rank sum test was used to analyze the differences in cNLR, cPLR and cLMR between groups, and graphs were drawn to view the trend of these parameters. All statistical analyses were performed using SPSS version 23.0 (SPSS, Inc.), and statistical significance was defined as *P < *.05.

## 3 RESULTS

### 3.1 Patient characteristics

The baseline patient, tumor, and treatment characteristics are presented in Table 1. The median follow‐up period was 57.6 months (range 4.4‐77.3). The ratio of males to females was 1.27:1. The mean age at diagnosis was 71.6 years, and most of the patients were not current smokers or drinkers. Sixty percent of the patients had stage I‐II disease, and the others had stage III‐IV disease (39.9%). The major locations of the primary tumors were the middle third (39.9%) and distal third (47.7%) of the esophagus. The mean tumor length was 4.7 cm, and 33.7% of the tumors were longer than 5 cm. There were 104 (53.9%) patients who underwent SIB‐IMRT, and 29 (15.0%) patients had CCRT. Only 10.4% of the patients had adverse events over grade 3.

**Table 1:** Baseline patient, tumor, and treatment characteristics

Characteristic	No. (%)
Age, mean (SD)	71.6 (8.1)
Sex	
Male	108 (56.0%)
Female	85 (44.0%)
Current smoker	
Yes	71 (36.8%)
No	122 (63.2%)
Alcohol	
Yes	28 (14.5%)
No	165 (85.5%)
BMI, mean (SD)	21.7 (3.1)
TNM stage	
I‐II	116 (60.1%)
III‐IV	77 (39.9%)
Tumor length, mean (SD)	4.7 (2.2)
Tumor location	
Proximal third	24 (12.4%)
Middle third	77 (39.9%)
Distal third	92 (47.7%)
Technique	
SIB	104 (53.9%)
SB	49 (25.4%)
CF	40 (20.7%)
CCRT	
Yes	29 (15.0%)
No	164 (85.0%)
Adverse event	
Grade ≥ 3	20 (10.4%)
Grade < 3	173 (89.6%)
Clinical Response	
cCR	39 (20.2%)
cPR	115 (59.6%)
cNR	39 (20.2%)

Abbreviations: BMI, body mass index; cCR, clinical complete response; CCRT, concurrent chemoradiotherapy; CF, conventional fractionated radiotherapy; cNR, clinical no response; cPR, clinical partial response; SB, sequential boost; SD, standard deviation; SIB, simultaneous integrated boost.

### 3.2 Peripheral blood cell count ratios during radiotherapy

**Figure 1:**
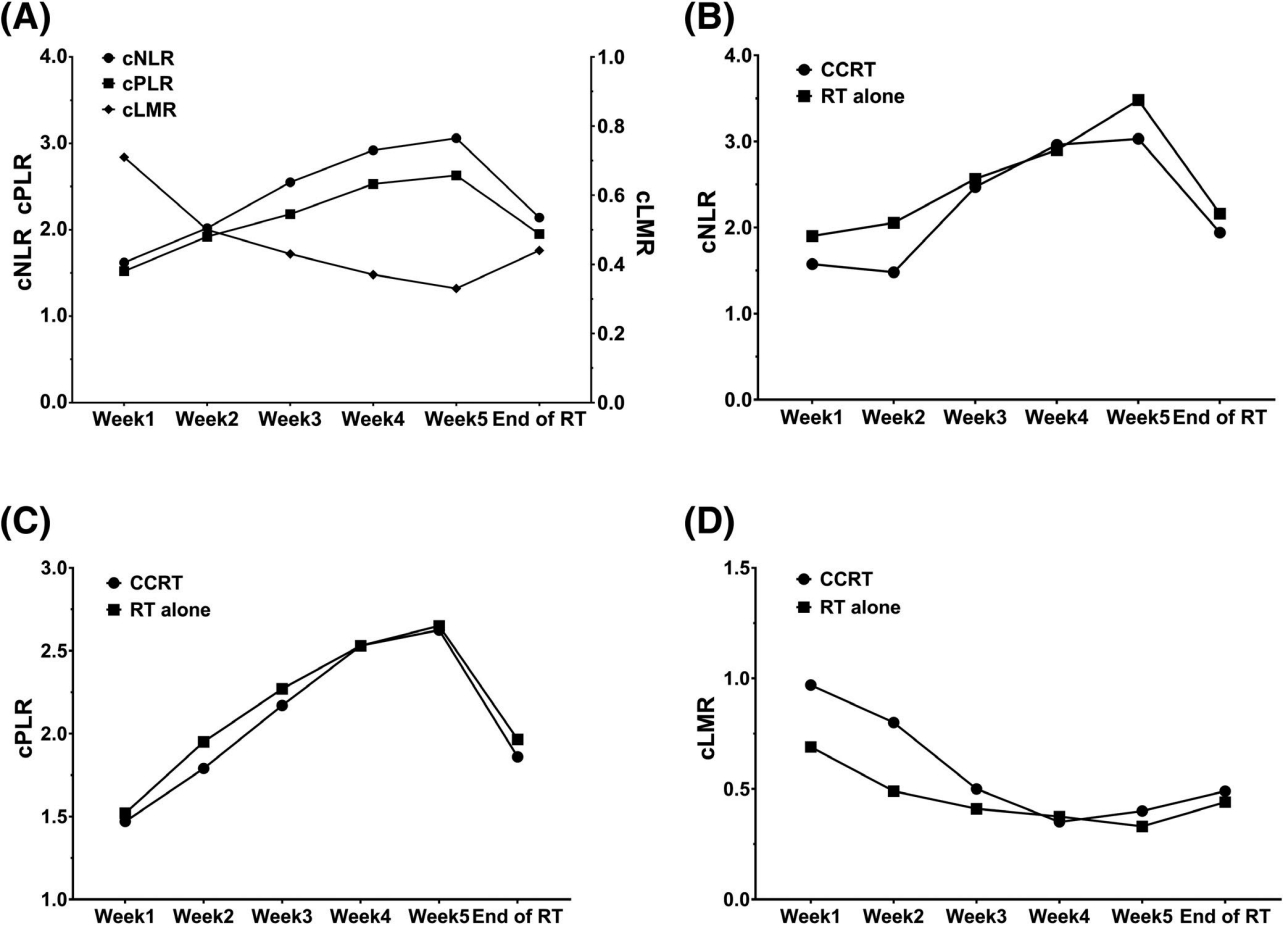
The median change rates of neutrophil‐to‐lymphocyte ratio (cNLR), platelet‐to‐lymphocyte ratio (cPLR), lymphocyte‐to‐monocyte ratio (cLMR) trend during radiotherapy (A); The median cNLR (B), cPLR (C), and cLMR (D) in concurrent chemoradiotherapy (CCRT) group and radiotherapy (RT) alone group trend during radiotherapy

During radiotherapy, the median cNLR and cPLR increased gradually every week, reaching the highest values at week 5 and then declining. In contrast, the median cLMR went down week by week and reached the lowest value at week 5 and then returned up (Figure 1A). The maximum weekly median cNLR and cPLR were 3.06 (95% CI = 2.76‐3.58) and 2.63 (95% CI = 2.33‐2.93), respectively. The minimum weekly median cLMR was 0.33 (95% CI = 0.30‐0.40). Most minimum or maximum values appeared in the fourth or fifth week of radiotherapy.

### 3.3 Association of peripheral blood cell count ratios with clinical response

We analyzed the relationship between clinicopathologic features, including peripheral blood cell count ratios, and clinical response (Table 2). Thirty‐nine (20.2%), 115 (59.6%), and 39 (20.2%) patients had cCR, cPR, and cNR, respectively. The treatment effective rate was 79.8%.

**Table 2:** Univariate and multivariate logistic regression analysis of clinical response

Variable	Univariate			Multivariate		
	OR	95% CI	*P* value	OR	95% CI	*P* value
Age	0.999	0.965‐1.034	.957			
Sex						
Male	1					
Female	1.067	0.610‐1.869	.819			
Current smoker						
Yes	1					
No	1.330	0.746‐2.374	.334			
Alcohol						
Yes	1					
No	1.149	0.522‐2.532	.730			
BMI	1.150	1.049‐1.262	.003*	1.200	1.041‐1.383	.012*
TNM stage						
I‐II	1			1		
III‐IV	0.228	0.121‐0.428	<.001*	0.185	0.061‐0.564	.003*
Tumor length						
<5 cm	1			1		
≥5 cm	0.350	0.189‐0.648	.001*	0.281	0.095‐0.834	.022*
Tumor location						
Proximal third	1					
Middle third	1.912	0.770‐4.746	.162			
Distal third	1.252	0.516‐3.036	.619			
Technique						
SIB	1					
SB	0.806	0.412‐1.576	.529			
CF	1.249	0.608‐2.565	.546			
CCRT						
Yes	1					
No	0.879	0.404‐1.915	.746			
Adverse event						
Grade ≥ 3	1			1		
Grade < 3	2.754	1.104‐6.869	.030*	11.566	2.056‐65.074	.005*
Week 1						
cNLR	0.713	0.551‐0.924	.011*	0.986	0.550‐1.770	.963
cPLR	0.574	0.413‐0.799	.001*	1.149	0.480‐2.749	.755
cLMR	1.585	1.086‐2.313	.017*	0.953	0.434‐2.095	.905
Week 2						
cNLR	0.629	0.509‐0.776	<.001*	0.919	0.566‐1.494	.735
cPLR	0.558	0.424‐0.735	<.001*	1.142	0.565‐2.308	.712
cLMR	2.000	1.254‐3.189	.004*	1.491	0.501‐4.440	.473
Week 3						
cNLR	0.458	0.364‐0.575	<.001*	0.975	0.585‐1.624	.922
cPLR	0.422	0.322‐0.554	<.001*	0.887	0.434‐1.813	.742
cLMR	2.207	1.405‐3.467	.001*	0.732	0.324‐1.653	.453
Week 4						
cNLR	0.373	0.292‐0.477	<.001*	0.607	0.373‐0.987	.044*
cPLR	0.299	0.220‐0.406	<.001*	0.522	0.286‐0.953	.034*
cLMR	3.397	2.084‐5.537	<.001*	1.794	0.780‐4.127	.169
Week 5						
cNLR	0.499	0.419‐0.595	<.001*	0.685	0.509‐0.924	.013*
cPLR	0.359	0.274‐0.470	<.001*	0.539	0.328‐0.886	.015*
cLMR	2.322	1.457‐3.699	<.001*	0.551	0.244‐1.245	.152
End of the treatment						
cNLR	0.553	0.459‐0.667	<.001*	0.728	0.497‐1.066	.103
cPLR	0.542	0.435‐0.677	<.001*	1.042	0.602‐1.804	.883
cLMR	2.864	1.686‐4.863	<.001*	1.197	0.533‐2.686	.664

Abbreviations: BMI, body mass index; CCRT, concurrent chemoradiotherapy; CF, conventional fractionated radiotherapy; CI, confidence interval; cLMR, change rate of lymphocyte‐to‐monocyte ratio; cNLR, change rate of neutrophil‐to‐lymphocyte ratio; cPLR, change rate of platelet‐to‐lymphocyte ratio; OR, odds ratio; SIB, simultaneous integrated boost, SB, sequential boost.

* Statistically significant.

The results of the univariate and multivariate ordinal logistic regression analyses for clinical response are shown in Table 2. Rates of change of peripheral blood cell count ratios each week, BMI, TNM stage, tumor length, and adverse event grade had significant associations with clinical responses on univariate analysis (all *P < *.050). cNLR at week 4 (OR = 0.607, 95% CI = 0.373‐0.987, *P* = .044), cPLR at week 4 (OR = 0.522, 95% CI = 0.286‐0.953, *P* = .034), cNLR at week 5 (OR = 0.685, 95% CI = 0.509‐0.924, *P* = .013), and cPLR at week 5 (OR = 0.539, 95% CI = 0.328‐0.886, *P* = .015) remained significant on multivariate analysis, along with BMI (OR = 1.200, 95% CI = 1.041‐1.383, *P* = .012), TNM stage (OR = 0.185, 95% CI = 0.061‐0.564, *P* = .003), tumor length (OR = 0.281, 95% CI = 0.095‐0.834, *P* = .022), and adverse event grade (OR = 11.566, 95% CI = 2.056‐65.074, *P* = .005). In summary, the elevation of the cNLR and cPLR at both weeks 4 and 5 implied poor clinical response. Age, sex, current smoking, alcohol use, tumor location, RT technique, and CCRT were not related to clinical response.

### 3.4 Association of peripheral blood cell count ratios with survival outcomes

By the end of follow‐up, the median overall survival was 33.5 months (95% CI = 27.0‐39.9). Cox regression analysis was performed for predictors of overall survival (Table 3). In the univariate analysis, we found that BMI, TNM stage, tumor length, tumor location, adverse event grade, cNLR, and cPLR were significant factors for prognosis (all *P* < .050). Then, the variables mentioned above were included in a multivariate Cox regression model for subsequent analysis. We finally demonstrated that elevated cNLR at week 4(HR = 1.181, 95% CI = 1.020‐1.369, *P* = .026), elevated cNLR at week 5 (HR = 1.144, 95% CI = 1.017‐1.288, *P* = .025), low BMI before treatment (HR = 0.940, 95% CI = 0.888‐0.996, *P* = .035), poor tumor stage (HR = 1.832, 95% CI = 1.218‐2.757, *P* = .004), tumor length over 5 cm (HR = 1.151, 95% CI = 1.047‐1.266, *P* = .004), tumor location in the proximal third of the esophagus (*P* < .001), and adverse event over grade 3(HR = 0.402, 95% CI = 0.237‐0.683, *P* = .001) were independent risk factors for poor OS. In contrast, other clinicopathological characteristics, cPLR and cLMR, did not have any significant prognostic influence.

**Table 3:** Univariate and multivariate Cox regression analysis of overall survival

Variable	Univariate			Multivariate			
	HR	95% CI	*P* value	HR	95% CI		*P* value
Age	1.011	0.989‐1.034	.336				
Sex							
Male	1						
Female	0.880	0.627‐1.235	.459				
Current smoker							
Yes	1						
No	0.743	0.526‐1.048	.090				
Alcohol							
Yes	1						
No	1.212	0.729‐2.016	.459				
BMI	0.916	0.866‐0.968	.002*	0.940	0.888‐0.996		.035*
TNM stage							
I‐II	1			1			
III‐IV	2.550	1.817‐3.579	<.001*	1.832	1.218‐2.757		.004*
Tumor length							
<5 cm	1						
≥5 cm	2.710	1.913‐3.840	<.001*	2.383	1.335‐4.254		.003*
Tumor location							
Proximal third	1		<.001*	1			<.001*
Middle third	0.248	0.146‐0.420	<.001*	0.224	0.124‐0.406		<.001*
Distal third	0.718	0.453‐1.136	.157	0.396	0.203‐0.772		.007*
Technique							
SIB	1		.553				
SB	1.056	0.714‐1.561	.786				
CF	0.803	0.507‐1.273	.352				
CCRT							
Yes	1						
No	1.314	0.799‐2.159	.282				
Adverse event							
Grade ≥ 3	1			1			
Grade < 3	0.468	0.284‐0.770	.003*	0.402	0.237‐0.683		.001*
Week 1							
cNLR	1.168	1.008‐1.353	.038*	1.088	0.827‐1.430		.547
cPLR	1.388	1.168‐1.649	<.001*	1.347	0.954‐1.900		.090
cLMR	0.929	0.738‐1.169	.529				
Week 2							
cNLR	1.148	1.032‐1.278	.011*	0.963	0.804‐1.153		.679
cPLR	1.234	1.074‐1.418	.003*	0.853	0.657‐1.108		.234
cLMR	0.939	0.703‐1.254	.670				
Week 3							
cNLR	1.356	1.206‐1.524	<.001*	1.153	0.948‐1.403		.155
cPLR	1.282	1.153‐1.424	<.001*	0.901	0.712‐1.140		.387
cLMR	0.939	0.711‐1.240	.657				
Week 4							
cNLR	1.287	1.175‐1.411	<.001*	1.181	1.020‐1.369		.026*
cPLR	1.249	1.152‐1.355	<.001*	0.993	0.840‐1.173		.932
cLMR	0.750	0.560‐1.004	.053				
Week 5							
cNLR	1.179	1.106‐1.256	<.001*	1.144	1.017‐1.288		.025*
cPLR	1.188	1.092‐1.292	<.001*	1.014	0.831‐1.236		.894
cLMR	0.800	0.590‐1.085	.151				
End of the treatment							
cNLR	1.134	1.042‐1.233	.003*	0.956	0.834‐1.095		.516
cPLR	1.145	1.024‐1.280	.017*	1.055	0.880‐1.265		.561
cLMR	0.809	0.603‐1.086	.159				

Abbreviations: BMI, body mass index; CCRT, concurrent chemoradiotherapy; CF, conventional fractionated radiotherapy; CI, confidence interval; cLMR, change rate of lymphocyte‐to‐monocyte ratio; cNLR, change rate of neutrophil‐to‐lymphocyte ratio; cPLR, change rate of platelet‐to‐lymphocyte ratio; HR, hazard ratio; SB, sequential boost; SIB, simultaneous integrated boost.

* Statistically significant.

For further analysis, we added CCRT as well as other significant factors into the multivariate Cox regression model again. The results showed that CCRT remained insignificant (Table S1). The model was statistically significant, and there is no significant collinearity among the parameters (Tables S2 and S3).

### 3.5 Comparison between CCRT and RT alone patients

The baseline clinical characteristics of patients in the CCRT group and RT alone group are shown in Table 4. The median age was 65 years (range 49‐80) in the CCRT group and 74 years (range 53‐87) in the RT alone group, showing a significant difference (*P* < .001). Additionally, patients who received CCRT had a higher BMI than patients who received RT alone (*P* = .044). Tumors in the CCRT group were all located in the middle and distal third of the esophagus, while in the RT alone group, 14.6% of the tumors were located in the proximal third of the esophagus (*P* = .002). Although there was no significant difference in the statistical distribution of TNM stage and tumor length, we still found that patients with stage Ⅰ‐Ⅱ disease and tumor length less than 5 cm mostly received RT alone. Sex, current smoking, alcohol use, technique, and adverse events showed no significant differences between the two groups.

**Table 4:** Baseline clinical characteristics of patients in CCRT group and RT alone group

Variable	CCRT group n (%)	RT alone group n (%)	*P* value
Age, median (range)	65 (49‐80)	74 (53‐87)	<.001*
Sex			
Male	18 (62.1)	90 (54.9)	.472
Female	11 (37.9)	74 (45.1)	
Current smoker			
Yes	10 (47.2)	61 (23.5)	.780
No	19 (52.8)	103 (76.5)	
Alcohol			
Yes	2 (6.9)	26 (15.9)	.329
No	27 (93.1)	138 (84.1)	
BMI, median (range)	22.7 (14.7‐28.7)	21.1 (14.7‐29.3)	.044*
TNM stage			
Ⅰ‐Ⅱ	15 (51.7)	101 (61.6)	.317
Ⅲ‐Ⅳ	14 (48.3)	63 (38.4)	
Tumor length			
<5cm	18 (62.1)	110 (67.1)	.599
≥5cm	11 (37.9)	54 (32.9)	
Tumor location			
Proximal third	0 (0.0)	24 (14.6)	.002*
Middle third	18 (62.1)	59 (36.0)	
Distal third	11 (37.9)	81 (49.4)	
Technique			
SIB	20 (69.0)	84 (51.2)	.100
SB	7 (24.1)	42 (25.6)	
CF	2 (6.9)	38 (23.2)	
Adverse event			
Grade ≥ 3	3 (10.3)	17 (10.3)	.997
Grade < 3	26 (89.7)	147(89.7)	

Abbreviations: BMI, body mass index; CCRT, concurrent chemoradiotherapy; CF, conventional fractionated radiotherapy; RT, radiotherapy; SB, sequential boost; SIB, simultaneous integrated boost

* Statistically significant.

During the treatment, the median cNLR and cPLR in the CCRT group were lower than those in the RT alone group, except at week 4, and the gap narrowed gradually (Figure 1B,C). At week 4, the median cNLR in the CCRT group became higher than that in the RT alone group, while the median cPLR was equal in the two groups. The trend of the cLMR was the opposite (Figure 1D). A detailed comparison is shown in Table 5. In addition to the cLMR at weeks 1 and 2 (*P* = .045, 0.029), no statistical difference was observed between the two groups.

**Table 5:** Median cNLR, cPLR and cLMR in CCRT group and RT alone group

	cNLR			cPLR			cLMR		
	CCRT	RT alone	*P* value	CCRT	RT alone	*P* value	CCRT	RT alone	*P* value
Week1	1.575	1.900	.819	1.470	1.520	.625	0.970	0.690	.045*
Week2	1.480	2.055	.245	1.790	1.950	.430	0.800	0.490	.029*
Week3	2.470	2.565	.609	2.170	2.270	.491	0.500	0.410	.322
Week4	2.960	2.900	.853	2.530	2.530	.526	0.350	0.375	.808
Week5	3.030	3.480	.282	2.625	2.650	.705	0.400	0.330	.616
End of RT	1.940	2.160	.536	1.860	1.965	.967	0.490	0.440	.417

Abbreviations: CCRT, concurrent chemoradiotherapy; cLMR, change rate of lymphocyte‐to‐monocyte ratio; cNLR, change rate of neutrophil‐to‐lymphocyte ratio; cPLR, change rate of platelet‐to‐lymphocyte ratio; RT, radiotherapy.

* Statistically significant.

**Figure 2:**
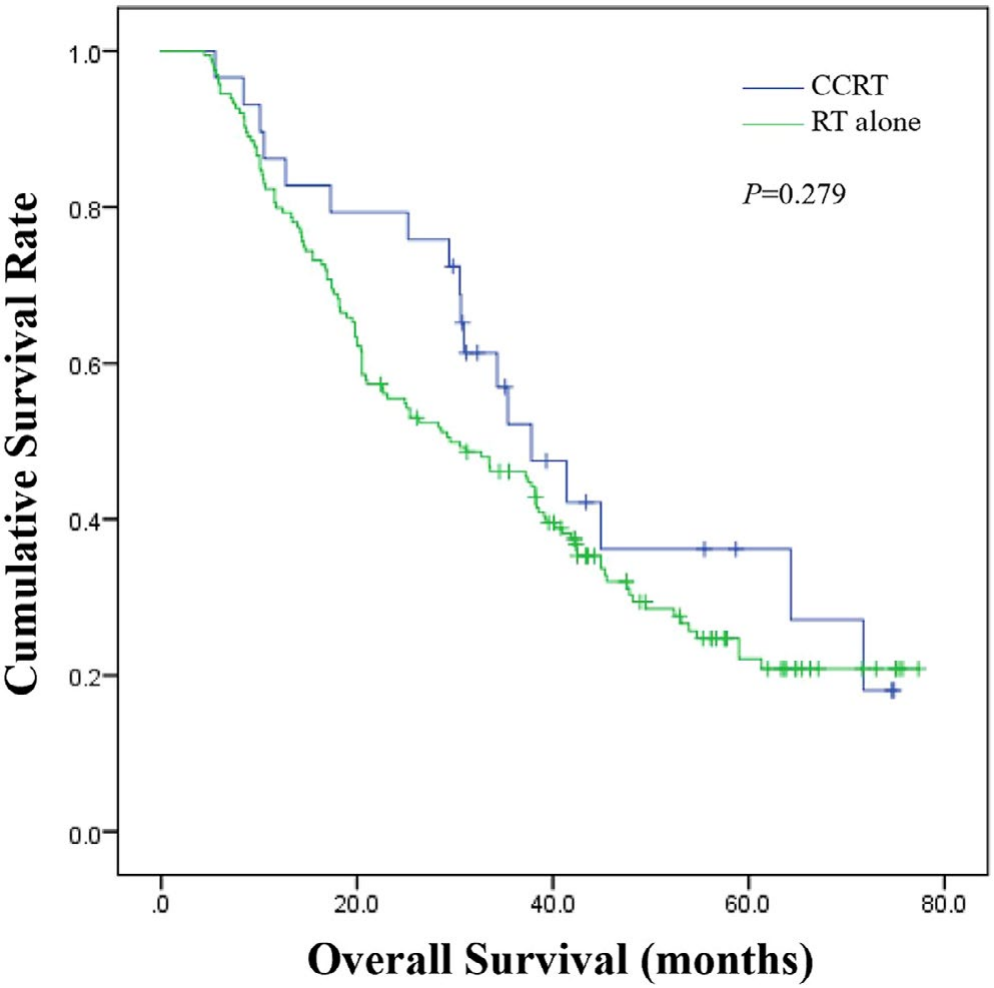
Overall survival of patients in concurrent chemoradiotherapy (CCRT) group versus radiotherapy (RT) alone group

The median OS of the CCRT group was 37.8 months (95% CI = 28.361‐47.239), while that of the RT alone group was 29.5 months (95% CI = 19.209‐39.791) (Figure 2). However, the log‐rank test showed that there was no statistical difference between the two groups (*P* = .279).

## 4 DISCUSSION

In this retrospective study, we identified the cNLR at weeks 4 and 5 during RT as prognostic biomarkers of OS for non‐surgical ESCC patients treated with radiotherapy. Meanwhile, patients with high cNLR and cPLR at weeks 4 and 5 had worse clinical responses. The peak values of cNLR and cPLR both appeared around week 5. Conversely, cLMR decreased to the minimum value at week 5 but had no predictive ability for either OS or clinical response.

For the past several years, inflammation has been investigated as a hallmark of cancer.^15^ Through modulation of the tumor microenvironment, inflammation can induce DNA damage, dysregulate the cell cycle, and lead to angiogenesis.^16^, ^17^ There are many biomarkers of the inflammatory response that have been reported to be related to tumor progression.^18^, ^19^ Among them, peripheral blood cell count ratios, such as NLR, PLR, and LMR, have received much attention due to their convenience and repeatability. However, though radiotherapy is the main treatment for non‐surgical ESCC patients, the relationship between the variation in peripheral blood cell count ratios during radiotherapy and prognosis remains unclear.

Circulating neutrophils contribute to tumor progression and invasiveness by secreting cytokines, vascular endothelial growth factor, and chemokines.^19^, ^20^, ^21^ As a component of the immune complex, lymphocytes, which can inhibit the proliferation and metastasis of tumors, play a crucial role in antitumor immunity.^22^ The NLR represents the balance between inflammatory and immune responses in peripheral blood. Previous studies have shown that a high pretreatment NLR is associated with a poor outcome in many solid tumors,^23^, ^24^, ^25^ while a low post‐treatment NLR can indicate a favorable prognosis.^26^ Radiotherapy can kill tumor cells directly or indirectly and stimulate inflammatory responses at the same time. Inflammation in radiotherapy is caused by the clearance of dying cells, leading to the modulation of the tumor microenvironment, which is a double‐edged sword in cancer treatment.^27^, ^28^, ^29^ It can boost immunity within certain limits but also cause tumor recurrence, radiation resistance, and severe side effects within high degree.^27^, ^30^, ^31^ Radiotherapy always has an effect on peripheral blood cells. Among leukocytopenias, lymphocytopenia is the most significant due to its high sensitivity to radiation,^32^, ^33^ which disrupts the balance between neutrophils and lymphocytes. cNLR can indicate variations in the inflammatory response and immunosuppression. Radiation with a single dose higher than 1 Gy might initiate inflammatory reactions, which gradually accumulate with increasing doses.^30^ This is in line with the weekly uptrend of NLR in our study and the gradually enhanced degree of variation. The reason why the maximum value appeared at week 5 and the value was reduced at the end of treatment may be due to the following reasons: (1) the use of antibiotics and glucocorticoids to alleviate side effects, such as radioactive esophagitis; and (2) the reduction of the tumor burden at the end of the treatment, which could decrease the degree of inflammatory reaction to some extent. Our research showed that the cNLR at weeks 4 and 5 had a negative correlation with the OS and clinical response of ESCC patients treated with radiotherapy. This indicates that, compared with the baseline value, the widest range of inflammation and immunosuppression during radiotherapy can be a predictive factor of clinical outcome.

Platelet count is another index of inflammation. Studies have shown that proinflammatory cytokines can facilitate the proliferation of megakaryocytes and increase platelet production.^34^, ^35^ Platelets can promote angiogenesis and tumor growth by producing cytokines, such as VEGF and transforming growth factor β.^4^, ^36^, ^37^ The significance of the correlation between PLR and tumor prognosis remains controversial. Some studies have shown that pretreatment PLR is an independent prognostic factor for tumor outcome,^38^, ^39^ while others report the opposite findings.^40^, ^41^ Decreases in platelet count also appear during radiotherapy but are not as significant as lymphocytopenia. Similar to cNLR, cPLR can also be an indicator to assess variations in the inflammatory response and immunosuppression. In our study, cPLR was upregulated with increased radiation dose and reached its peak at week 5. This was consistent with the uptrend of inflammation during radiotherapy, and the value at the end of the treatment was under the influence of drug therapy and the decreased tumor load. Elevated cPLR at weeks 4 and 5 was significantly associated with poor clinical outcome. These results reconfirm the close relationship between the clinical response of RT and the maximum degree of variation in inflammation and immunoreaction in ESCC patients. Regarding prognosis, cPLR each week was a statistically significant variable in the univariate analysis but was rejected after the multivariate analysis, which meant that the predictive ability of cPLR was weaker than that of cNLR.

Monocytes also participate in tumor development. Monocytes can differentiate into tumor‐associated macrophages (TAMs), which promote tumor progression by secreting growth factors and cytokines.^42^, ^43^ Previous studies have suggested that baseline LMR, another indicator of inflammation and immune status, is a prognostic factor in nasopharyngeal carcinoma,^44^ gastric cancer,^45^ and hepatocellular carcinoma.^46^ However, some reports do not support this view.^47^ According to the present study, LMR decreased weekly, and the minimum value appeared at week 5. This is in accordance with the dramatic reduction in lymphocytes during RT in ESCC patients. The extent of LMR change showed no connection with either clinical responses or OS. This may be due to the insufficiency of samples or the low absolute value of cLMR, which may affect the effectiveness of statistical test.

Chemotherapy has an impact on peripheral blood cell count and has shown some common patterns. As patients received chemotherapy at week 1, chemotherapeutic drug‐induced agranulocytosis began to appear, which caused the patients in the CCRT group to have a lower cNLR, lower cPLR, and higher cLMR than those in the RT alone group. During the intermittent period of chemotherapy, peripheral blood cells, especially granulocytes, began to recover gradually, so the gap narrowed. The two lines began to cross at week 4. This may be due to several reasons: (a) the removal of tumor cells by chemotherapy leads to a stronger inflammatory response; and (b) chemotherapy has a certain radiosensitization effect, leading to an increased inflammatory response. As to week 5, with the second cycle of chemotherapy, cNLR and cPLR became lower and cLMR became higher again in the CCRT group. This was consistent with the side effects of chemotherapeutic drugs. Although there was no significant difference between the cNLR, cPLR, and cLMR each week in the two groups except the cLMR at weeks 1 and 2, we still found a difference in the median. This may be due to the statistical deviation caused by the large difference in sample size between the two groups.

It is widely accepted that CCRT is one of the standard treatments for non‐surgical esophageal cancer. In our study, most of the patients were elderly and with early stage. Toxicity and patient tolerance should be taken into consideration. Studies have shown that elderly EC patients have poor tolerance to the short‐term and long‐term toxicity after CCRT, especially for patients older than 75 years.^48^, ^49^, ^50^ These studies are similar to our treatment options. In the CCRT group, patients were younger and had better nutritional status. Elderly patients with early‐stage tumors and small tumor length mostly received radical RT. This is consistent with the guidelines. Studies have shown that CCRT is an important prognostic factor of EC patients.^51^ However, other researchers hold the view that elderly patients cannot achieve better overall survival from CCRT,^52^, ^53^ which in agreement with our findings. Although patients in the CCRT groups have longer median survival than patients in the RT group, no statistical significance was observed.

The highlight of our study is that we assessed the association of dynamic changes in peripheral blood cell count ratios with clinical response and OS for non‐surgical ESCC patients treated with radiotherapy and identified the optimal time window for evaluation. We calculated the rate of change in the ratios to evaluate the degree of variation, with the exception of the influence of the baseline inflammatory and immune state.

There are some limitations in our study. First, this is a retrospective study at a single center, and the evaluation of the clinical responses was based on barium radiography and CT but not PET/CT or biopsy. In addition, although the process of patient selection was rigorous, we could not completely rule out all factors that might potentially impact peripheral blood cell count ratios. Third, we did not conduct a further analysis to confirm the cutoff value of these parameters, and progression‐free survival was not taken into consideration because of the lack of accurate data. It is obvious that a large‐sample and multicenter perspective study should be conducted in the future to further confirm these results.

In conclusion, we demonstrated that the cNLR at weeks 4 and 5 during RT had a significant negative correlation with the OS and clinical responses of non‐surgical ESCC patients treated with radiotherapy, while an elevated cPLR at weeks 4 and 5 was only related to poor clinical response. These findings can be used as a basis for the dynamic evaluation of patient clinical responses and prognosis during radiotherapy through simple and repeatable biomarkers.

## CONFLICT OF INTEREST

The authors stated that there are no conflicts of interest regarding the publication of this article.

## Supporting information

Supplementary Material
