# Supplementary material for: Peripheral blood cell count ratios are predictive biomarkers of clinical response and prognosis for non‐surgical esophageal squamous cell carcinoma patients treated with radiotherapy
Source: J Clin Lab Anal. 2020 Jul 17;34(10):e23468. doi: 10.1002/jcla.23468 (PMC7595892; doi:10.1002/jcla.23468)
Supplement: Supplementary file 1 — Supplementary Material [file JCLA-34-e23468-s001.docx]

**Supplement 1.** Multivariable Cox regression analysis of overall survival including CCRT

| **Variable** | **Multivariate** | | |
| --- | --- | --- | --- |
|  | **HR** | **95% CI** | ***P* value** |
| BMI | 0.952 | 0.898-1.010 | .104 |
| TNM stage |  |  |  |
| Ⅰ-Ⅱ | 1 |  |  |
| Ⅲ-Ⅳ | 1.905 | 1.261-2.877 | .002* |
| Tumor length |  |  |  |
| ＜5cm | 1 |  |  |
| ≥5cm | 2.568 | 1.422-4.639 | .002* |
| Tumor location |  |  |  |
| Proximal third | 1 |  | ＜.001* |
| Middle third | 0.242 | 0.133-0.442 | ＜.001* |
| Distal third | 0.383 | 0.195-0.750 | .005* |
| CCRT |  |  |  |
| Yes | 1 |  |  |
| No | 1.613 | 0.892-2.916 | .114 |
| Adverse event |  |  |  |
| Grade≥3 | 1 |  |  |
| Grade < 3 | 0.392 | 0.230-0.668 |  |
| Week 1 |  |  |  |
| cNLR | 1.098 | 0.838-1.440 | .496 |
| cPLR | 1.319 | 0.929-1.872 | .121 |
| Week 2 |  |  |  |
| cNLR | 0.987 | 0.818-1.192 | .896 |
| cPLR | 0.859 | 0.661-1.117 | .258 |
| Week 3 |  |  |  |
| cNLR | 1.150 | 0.944-1.401 | .165 |
| cPLR | .905 | 0.717-1.143 | .402 |
| Week 4 |  |  |  |
| cNLR | 1.145 | 0.984-1.333 | .080 |
| cPLR | 1.004 | 0.851-1.184 | .965 |
| Week 5 |  |  |  |
| cNLR | 1.165 | 1.034-1.313 | .012* |
| cPLR | 1.022 | 0.840-1.243 | .829 |
| End of the treatment |  |  |  |
| cNLR | 0.966 | 0.843-1.108 | .625 |
| cPLR | 1.011 | 0.836-1.223 | .910 |

Abbreviations: CCRT, concurrent chemoradiotherapy; HR, hazard ratio; CI, confidence interval; BMI, body mass index; cNLR, change rate of neutrophil-to-lymphocyte ratio; cPLR, change rate of platelet-to-lymphocyte ratio; cLMR, change rate of lymphocyte-to-monocyte ratio. *Statistically significant.

**Supplement 2.** Collinearity Statistics of multivariable Cox regression analysis of overall survival including CCRT

| **Variable** | **Tolerance** | **VIF** |
| --- | --- | --- |
| BMI | 0.918 | 1.089 |
| TNM stage | 0.769 | 1.300 |
| Tumor length | 0.518 | 1.931 |
| Tumor location | 0.580 | 1.725 |
| CCRT | 0.945 | 1.059 |
| Adverse event | 0.958 | 1.044 |
| Week 1 |  |  |
| cNLR | 0.428 | 2.339 |
| cPLR | 0.350 | 2.856 |
| Week 2 |  |  |
| cNLR | 0.444 | 2.252 |
| cPLR | 0.396 | 2.525 |
| Week 3 |  |  |
| cNLR | 0.312 | 3.200 |
| cPLR | 0.278 | 3.594 |
| Week 4 |  |  |
| cNLR | 0.339 | 2.951 |
| cPLR | 0.291 | 3.441 |
| Week 5 |  |  |
| cNLR | 0.402 | 2.489 |
| cPLR | 0.312 | 3.204 |
| End of the treatment |  |  |
| cNLR | 0.427 | 2.343 |
| cPLR | 0.365 | 2.739 |

Abbreviations: CCRT, concurrent chemoradiotherapy; VIF, variance inflation factor; BMI, body mass index; cNLR, change rate of neutrophil-to-lymphocyte ratio; cPLR, change rate of platelet-to-lymphocyte ratio; cLMR, change rate of lymphocyte-to-monocyte ratio. *Statistically significant.

**Supplement 3.** Omnibus Tests of Model Coefficients

| **-2 Log Likelihood** | **Overall (score)** | | | **Change From Previous Block** | | |
| --- | --- | --- | --- | --- | --- | --- |
|  | **Chi-square** | **df** | **Sig.** | **Chi-square** | **df** | **Sig.** |
| 1145.900 | 136.299 | 19 | .000 | 129.789 | 19 | .000 |

Abbreviations: df, degrees of freedom; Sig, significance.
